# Research of Prostate Cancer Urinary Diagnostic Biomarkers by Proteomics: The Noteworthy Influence of Inflammation

**DOI:** 10.3390/diagnostics13071318

**Published:** 2023-04-01

**Authors:** Elisa Bellei, Stefania Caramaschi, Giovanna A. Giannico, Emanuela Monari, Eugenio Martorana, Luca Reggiani Bonetti, Stefania Bergamini

**Affiliations:** 1 Department of Surgery, Medicine, Dentistry and Morphological Sciences with Transplant Surgery, Oncology and Regenerative Medicine Relevance, Proteomic Lab, University of Modena and Reggio Emilia, 41124 Modena, Italy; elisa.bellei@unimore.it (E.B.); emanuela.monari@unimore.it (E.M.); 2 Department of Medical and Surgical Sciences for Children and Adults, University of Modena and Reggio Emilia, AOU Policlinico di Modena, 41124 Modena, Italy; stefania.caramaschi@unimore.it (S.C.); luca.reggianibonetti@unimore.it (L.R.B.); 3 Department of Pathology, Microbiology and Immunology, Vanderbilt University Medical Center, Nashville, TN 37232, USA; giovanna.giannico@vumc.org; 4 Division of Urology, New Civilian Hospital of Sassuolo, 41049 Modena, Italy; eugeniomartorana@gmail.com

**Keywords:** prostate cancer, benign prostatic hyperplasia, diagnostic biomarkers, proteomics, urine, inflammation

## Abstract

Nowadays, in the case of suspected prostate cancer (PCa), tissue needle biopsy remains the benchmark for diagnosis despite its invasiveness and poor tolerability, as serum prostate-specific antigen (PSA) is limited by low specificity. The aim of this proteomic study was to identify new diagnostic biomarkers in urine, an easily and non-invasively available sample, able to selectively discriminate cancer from benign prostatic hyperplasia (BPH), evaluating whether the presence of inflammation may be a confounding parameter. The analysis was performed by two-dimensional gel electrophoresis (2-DE), mass spectrometry (LC-MS/MS) and Enzyme-Linked Immunosorbent Assay (ELISA) on urine samples from PCa and BPH patients, divided into subgroups based on the presence or absence of inflammation. Significant quantitative and qualitative differences were found in the urinary proteomic profile of PCa and BPH groups. Of the nine differentially expressed proteins, only five can properly be considered potential biomarkers of PCa able to discriminate the two diseases, as they were not affected by the inflammatory process. Therefore, the proteomic research of novel and reliable urinary biomarkers of PCa should be conducted considering the presence of inflammation as a realistic interfering element, as it could hinder the detection of important protein targets.

## 1. Introduction

To date, prostate cancer (PCa) is the second most common male malignancy, according to the estimated number of incident cases worldwide [1]. An increase in the number of new cases is expected in the coming years [2]. Rising incidence is secondary to longer life expectancy, population preventive strategies, and serum prostate-specific antigen (PSA) screening [3]. Presently, PCa appears as the fifth most common global cause of death from cancer in men [4]. Survival is 88% at 5 years after diagnosis and is increasing over time [5]. The pathogenesis of PCa is multifactorial due to the interaction of different risk factors, such as age, geographic area, familiarity, lifestyle, environmental agents, and genetic factors [6], while its etiology is not yet fully understood.

Current screening strategies for PCa diagnosis include prostatic digital rectal examination (DRE) and PSA measurement. However, neither can reliably differentiate a benign prostate condition from a cancerous one. PSA is an organ-specific and not a tumor-specific marker. A high PSA level can also be found in other non-malignant prostate conditions, such as benign prostatic hyperplasia (BPH) or prostatitis [7], and after prostate instrumentation or cystoscopy. Therefore, at present, the diagnostic gold standard is prostate needle biopsy (PBx), which is performed following an abnormal DRE and/or PSA. Unfortunately, this is an invasive technique associated with various side effects. Furthermore, about two-thirds of biopsies performed following elevated PSA were unneeded [8], underscoring the low specificity of this marker, which can lead to over-diagnosis of PCa and unnecessary treatment [9].

Hence, there is a pressing need for selective and noninvasive biomarkers for an accurate diagnosis and management of PCa. In this regard, urine is one of the most attractive biofluids in clinical proteomics, as it can be collected non-invasively, easily, inexpensively and in the large amount [10]. Moreover, compared to other biological specimens, such as plasma and serum, urine has a quite simple composition, as it contains a relatively lower number of proteins. This characteristic is a great advantage in the proteomic analysis [11]. Beyond urine, another encouraging and emerging strategy to deepen the knowledge of PCa is liquid biopsy, as it represents the tumor microenvironment, thus allowing overall information for the disease diagnosis, prognosis, monitoring and treatment [12]. In this regard, it has recently been reported that BRCA germline mutations have important implications in assessing the risk of developing PCa, as well as in the prognosis and treatment of the disease, proving their relevance in the clinical setting [13].

In the last few years, growing numbers of promising biomarkers have been described for different urological tumors, such as bladder cancer, renal cell carcinoma, and PCa, by urine-based proteomics studies [14,15]. Therefore, this approach could have the potential to limit tissue biopsy sampling, thus reducing the number of patients undergoing PBx.

Inflammation is an inherent and important component of cancer disease, with a substantial impact on studies aimed at the discovery and validation of biomarkers. Despite its crucial aspect, inflammation is frequently overlooked in most cancer biomarker studies [16,17]. Notably, our prior proteomic study conducted on serum samples from PCa and BPH patients has focused on the presence of inflammation, demonstrating that it can be a confounding parameter for protein biomarker discovery [18].

In the present study, new potential urinary diagnostic biomarkers of PCa were searched by two-dimensional gel electrophoresis (2-DE) associated with mass spectrometry analysis (LC-MS/MS). Significantly, the urinary proteomic profiles of PCa and BPH were compared, considering the presence or absence of inflammation.

## 2. Materials and Methods

### 2.1. Patients Selection and Classification

Patient enrollment was conducted at the Urology Department of the University Hospital Policlinico di Modena (Italy). All procedures followed in the study were approved by the Provincial Ethics Committee of Modena, Italy (Project identification code 57/08). The enrolled subjects provided written informed consent to participate in the study, which was conducted according to the ethical principles of the Helsinki Declaration (2013, last edition).

Inclusion criteria were age between 58 and 81 years, elevated serum PSA, and palpable lesions on DRE in patients who underwent PBx. Exclusion criteria were the presence of systemic diseases, renal disorders, diabetes, proteinuria from a routine clinical laboratory test, significant clinical events occurring within 6 months of enrollment, hormonal treatment, radiotherapy, and chemotherapy.

Patients with histopathologic diagnosis of PCa and BPH were included in the study in two separate groups: the PCa group (comprising all patients diagnosed with PCa) and the BPH group (comprising all patients diagnosed with BPH) [19]. Each group was further divided into two subgroups considering the absence or presence of inflammation, assessed during histological examination by the occurrence of pathological infiltration of the prostatic tissue by inflammatory cells, namely aggregations of lymphocytes and plasma cells. All slides were stained with hematoxylin and eosin (H&E) and were re-reviewed and graded by a pathologist according to the 2014 Modified Gleason System [20]. Therefore, the urinary proteome analysis was performed on six groups: (1) all cases of PCa (PCa group, *n* = 30), (2) PCa without inflammation (noI-PCa group, *n* = 10), (3) PCa with inflammation (I-PCa group, *n* = 20), (4) all cases of BPH (BPH group, *n* = 30), (5) BPH without inflammation (noI-BPH group, *n* = 11), and (6) BPH with inflammation (I-BPH group, *n* = 19).

### 2.2. Urinary Samples Preparation

Morning urine samples (10 mL, midstream) were collected before the biopsy. After centrifugation (800× *g*, 4 °C, 10 min) to remove any cellular debris and contamination, they were stored in aliquots at −80 °C for proteomic analysis. Urine samples were then pooled for each group (4 mL/pool), desalted, and concentrated to a final volume of 100/120 μL with Desalting Spin Columns (cut-off 3 kDa MW, Amicon Ultra-4, Merck Millipore, Milan, Italy) following the manufacturer’s instructions.

### 2.3. Proteins Quantification

Protein content from each concentrated pool was quantified by the Bradford method [21] using the Protein Assay Dye Reagent (Bio-Rad Laboratories, Hercules, CA, USA) and bovine serum albumin (Sigma-Aldrich, Milan, Italy) as colorimetric reagent and calibration standard, respectively. Optical density (OD) reading was performed at 595 nm using a microplate reader (Multiskan^TM^ FC, Thermo Fisher Scientific, Waltham, MA, USA).

### 2.4. Proteomic Analysis

The 2-DE analysis was performed on concentrated urinary pools. The proteins of each group (80 µg) were solubilized with rehydration buffer (6 M urea, 2 M thiourea, 4% 3-[(3-Cholamidopropyl)-dimethylammonio]-propane-sulfonate, CHAPS, 25 mM dithiothreitol, 0.2% ampholytes) and subjected to the first-dimension separation using 17 cm Immobilized pH Gradient strips (IPG strip, Bio-Rad Laboratories, Hercules, CA, USA), narrow pH range 4–7, and 8–16% polyacrylamide gradient gels in the second-dimension separation, as previously fully described [18].

After 2-DE, gels were stained with a silver nitrate protocol [22], then the gel images were acquired by a calibrated densitometer (series GS-800, Bio-Rad Laboratories, Hercules, CA, USA). The PDQuest 2-D Image analysis software program, version 7.3.1 (Bio-Rad Laboratories, Hercules, CA, USA) (accessed on 4 May 2022), was used to evaluate the spot intensity as OD (mean value) × area (mm^2^). The difference in protein expression (fold-change) among the groups was obtained by the ratio between the values of spot intensity: variations ≥ 1.5 were considered statistically significant.

The different spots were manually removed from the gels and subjected to “in-gel trypsin digestion” for subsequent protein identification by LC-MS/MS, using a 1200 Nano HPLC/Chip microfluidic device (Agilent Technologies Inc., Santa Clara, CA, USA) associated with a 6520 Accurate-Mass ElectroSprayIonization-Quadrupole-Time-of-Flight mass spectrometer (ESI-Q-ToF, Agilent Technologies Inc., Santa Clara, CA, USA), as previously described [22]. Triptych digests were first resuspended in 10 μL of 3% acetonitrile (ACN)/0.1% formic acid (FA), then MS analysis was performed at a flow rate of 0.4 μL/min with a mobile phase composed of 95% ACN/5% water/0.1% FA, using a Chip enrichment column (Zorbax C18, 4 mm × 5 μm i.d., Agilent Technologies Inc., Santa Clara, CA, USA) and a separation column (Zorbax C18, 43 mm × 75 μm i.d., Agilent Technologies Inc., Santa Clara, CA, USA), with nitrogen as the nebulizer gas.

Protein characterization was performed using the SwissProt database and the search engine Mascot MS/MS Ion Search (accessed on 13 May 2022), specifying the following parameters: species Homo sapiens (Human), two possibilities of trypsin failure, peptide tolerance ± 20 ppm, MS/MS error tolerance ± 0.1 Da, carbamidomethylation of cysteines as fixed modifications and methionine oxidation as variable modifications.

### 2.5. Enzyme-Linked Immunosorbent Assay (ELISA)

The commercial Human Prostaglandin D Synthase (Lipocalin-Type) ELISA kit (BioVendor, TEMA Ricerca S.r.l., Castenaso, BO, Italy) It was used to validate and quantify Prostaglandin-H2 D-isomerase (PTGDS) in urine samples of patients from the four subgroups. Diluted urine samples (1:100) were analyzed in duplicate following the manufacturer’s recommendations. The absorbance of the final product was measured at 450 nm using a microplate reader (Multiskan FC, Thermo Fisher Scientific), and protein concentration was calculated from a standard curve generated by the stock solution furnished with the kit.

### 2.6. Statistical Analysis

The patient’s age and PSA levels are reported as the median. Total protein content in each group and PTGDS concentrations are provided as mean ± standard deviation (SD). The statistical analysis was carried out using the parametric Student’s *t*-test, considering *p*-values ≤ 0.05 as statistically significant.

## 3. Results

### 3.1. Histological Examination

**Figure 1:**
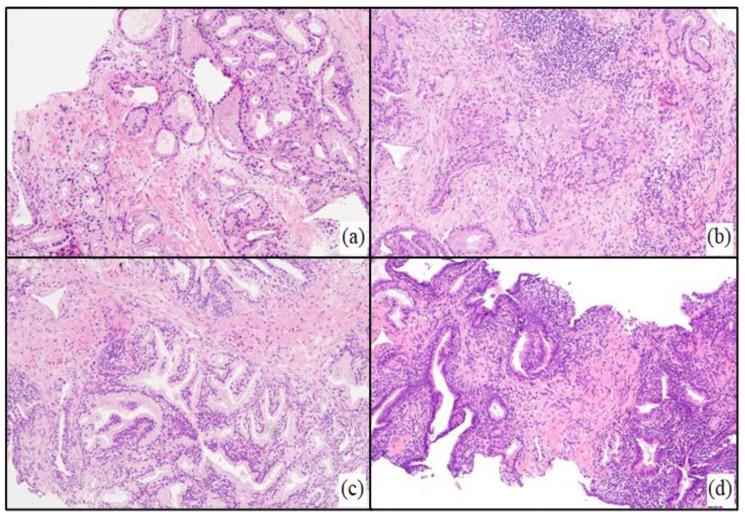
Representative histological slides displaying the different tissue conditions for the four subgroups: (**a**) noI-PCa; (**b**) I-PCa; (**c**) noI-BPH; (**d**) I-BPH.

PCa was characterized by atypical glands or nests of irregular epithelial cells with amphophilic cytoplasm, enlarged hyperchromatic nuclei, and prominent nucleoli. BPH was diagnosed when were observed increases in epithelial and/or stromal cells and typical nodular and expansive growth without evidence of atypia. PCa and BPH patients were stratified into 4 subgroups based on the absence or presence of inflammation, determined by the presence of inflammatory cells infiltrating the prostate tissue. Representative H&E slides for the different tissue conditions are shown in Figure 1.

### 3.2. Clinical Data

The median age of the enrolled patients was 68 years (range 59–73) for the PCa group and 68 years (range 59–81) for the BPH group, denoting no significant differences between the two groups, as well as among the relative subgroups. Within noI-PCa, five patients showed Grade Group (G) < 7 and 5 patients G ≥ 7. Within I-PCa, nine patients showed G < 7, and 11 cases showed G ≥ 7. PSA values were significantly higher in PCa vs. BPH (*p* = 0.03) and in noI-PCa vs. noI-BPH (*p* = 0.02) (Table 1).

### 3.3. Protein Concentration

The spectrophotometric quantification of urinary protein content is illustrated in Table 2. PCa groups showed a significantly lower total protein concentration than BPH groups, regardless of inflammation (PCa vs. BPH, *p* < 0.001; noI-PCa vs. noI-BPH, *p* ≤ 0.05; I-PCa vs. I-BPH, *p* ≤ 0.05). When comparing the same group with and without inflammation, total protein content was found to be significantly higher in I-BPH vs. noI-BPH (*p* ≤ 0.05), while the result was not significantly different in Pca (*p* > 0.05).

### 3.4. Proteomic Comparisons

**Figure 2:**
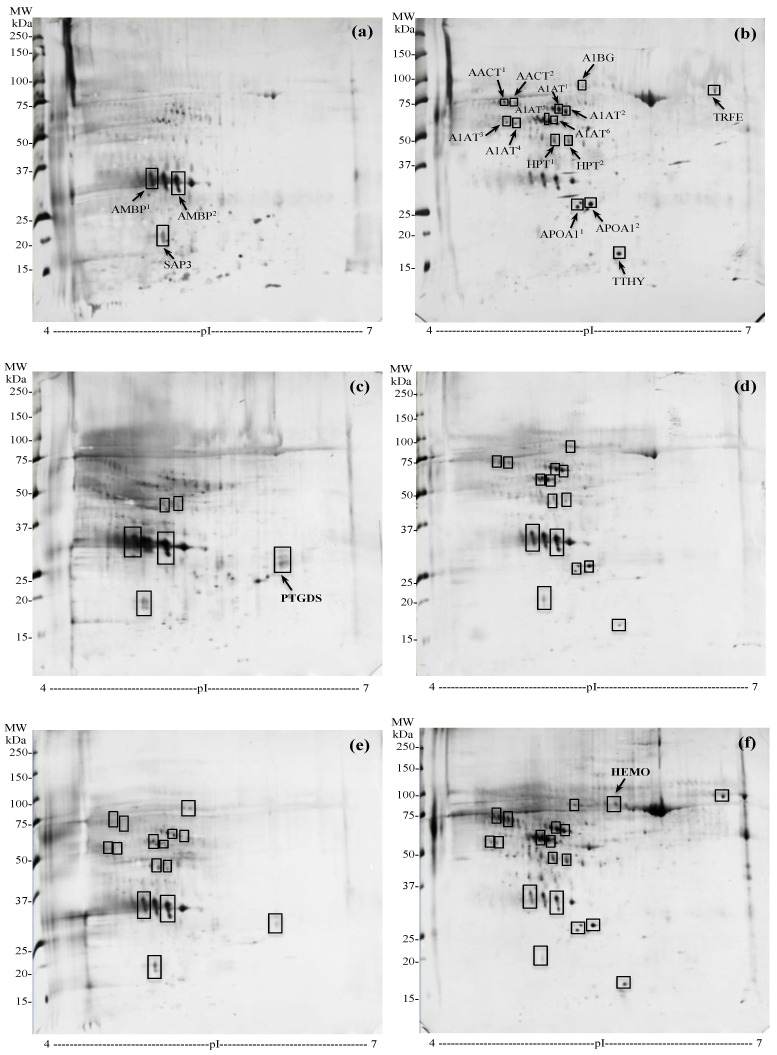
Representative urinary 2D gels; (**a**) PCa group (all PCa patients with and without inflammation); (**b**) BPH group (all BPH patients with and without inflammation); (**c**) noI-PCa group (PCa patients without inflammation); (**d**) noI-BPH group (BPH patients without inflammation); (**e**) I-PCa group (PCa patients with inflammation); (**f**) I-BPH group (BPH patients with inflammation). The significantly different spots are enclosed in rectangles, and in panels (**a**–**c**,**f**) are included the protein entry names, corresponding to those reported in Table 3. AMBP, Alpha-1-microglobulin; SAP3, Ganglioside GM2 activator; AACT, Alpha-1-antichymotrypsin; A1BG, Alpha-1-beta-glicoprotein; TRFE, Serotransferrin; A1AT, Alpha-1-antitrypsin; HPT, Haptoglobin; APOA1, Apolipoprotein A1; TTHY, Transthyretin; PTGDS, Prostaglandin-H2 D-isomerase; HEMO, Hemopexin.

The urinary protein maps obtained by 2-DE analysis for each group are shown in Figure 2.

#### 3.4.1. Urinary Protein Expression in PCa and BPH Regardless Inflammation

The comparison of the urinary proteomic profiles of PCa and BPH, regardless of the presence or absence of inflammation, showed 18 differentially expressed protein spots, corresponding to 9 unique proteins. Specifically, three spots, two of which related to Alpha-1-microglobulin (AMBP^1^ and AMBP^2^), and one identified as Ganglioside GM2 activator (SAP3) were increased in PCa (Figure 2a) compared to BPH (Figure 2b). In the same comparison, 15 spots resulted decreased: Alpha-1-antichymotrypsin (spots AACT^1^ and AACT^2^), Alpha-1-beta-glycoprotein (A1BG), Serotransferrin (TRFE), six isoforms of Alpha-1-antitrypsin (A1AT^1^-A1AT^6^), two spots recognized as Haptoglobin (HPT^1^ and HPT^2^), two spots corresponding to Apolipoprotein A1 (APOA1^1^ and APOA1^2^), and Transthyretin (TTHY).

#### 3.4.2. Urinary Protein Expression in Specimens without Inflammation

Similar to the previous comparison, AMBP^1^, AMBP^2^, and SAP3 were overexpressed, while AACT, A1BG, A1AT, and APOA1 were decreased in noI-PCa (Figure 2c) vs. noI-BPH (Figure 2d). Otherwise, no expression difference was revealed for HPT and TTHY, while TRFE was not detectable. Noteworthy, a novel spot was significantly detected in noI-PCa, identified as PTGDS.

#### 3.4.3. Urinary Protein Expression in PCa with and without Inflammation

AMBP^1^, AMBP^2,^ SAP3, AACT^1^ and AACT^2^ spots were decreased, while A1BG, A1AT and HPT were increased in I-PCa (Figure 2e) vs. noI-PCa (Figure 2c). TRFE, APOA1, and TTHY were not detectable. Remarkably, PTGDS was found to be downregulated in the presence of inflammation.

#### 3.4.4. Urinary Protein Expression in BPH with and without Inflammation

AMBP^1^, AMBP^2^ and SAP3 were decreased, while AACT, A1BG, TRFE, A1AT, HPT, and TTHY resulted in an increase in I-BPH (Figure 2f) compared to noI-BPH (Figure 2d). APOA1 and PTGDS showed no significant expression difference. Additionally, a new protein spot, identified as Hemopexin (HEMO), was revealed only in I-BPH.

The values of fold-change in expression obtained by each comparison are reported in Table 3, as well as the complete list of proteins identified by LC-MS/MS analysis. All proteins presented the highest ion scores obtained with the MASCOT search engine (ranging from 2686 to 51), indicating the observed correspondence between the experimental data and the theoretical data. Moreover, the number of significant sequences, that is, the number of significant peptides that matched the identified proteins, were at least >2, and the protein coverage, namely the percentage of amino acids sequenced for each detected protein, ranged from a minimum of 13% up to 66%.

**Figure 3:**
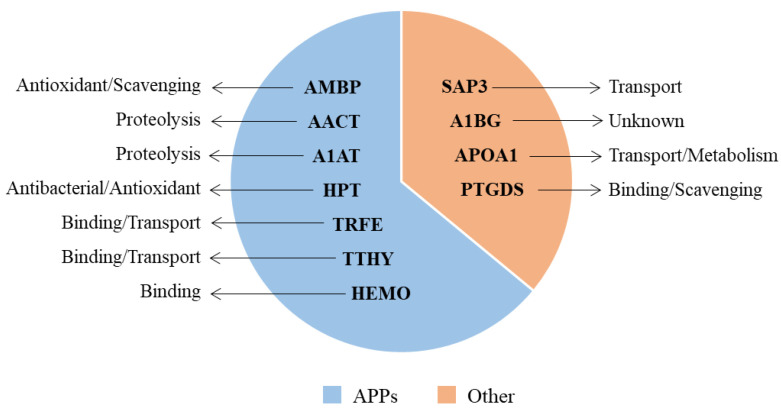
Principal roles of the differentially expressed proteins. The majority (64%) were acute-phase response proteins (APPs) involved in various biological processes, while the others (36%) were proteins with similar or different molecular functions.

In addition, were pointed out the main functions of each protein (Figure 3). As evidenced, most of the detected proteins (64%) were acute-phase proteins (APPs). However, these proteins play other roles, including antioxidant, radical scavenging, and tissue repair (AMBP), proteolysis (AACT, A1AT), antioxidant and antibacterial activity (HPT), iron binding and transport (TRFE), hormone binding and transport (TTHY), heme and metal ion binding (HEMO). Other proteins (36%) were involved in further biological actions, namely lipid transport (SAP3), steroid metabolism and cholesterol transport (APOA1), binding of lipophilic molecules and scavenging of harmful hydrophobic constituents (PTGDS), as inferred by the UniProtKB and The Human Protein Atlas databases (accessed on 30 May 2022).

### 3.5. PTGDS Quantification by ELISA

**Figure 4:**
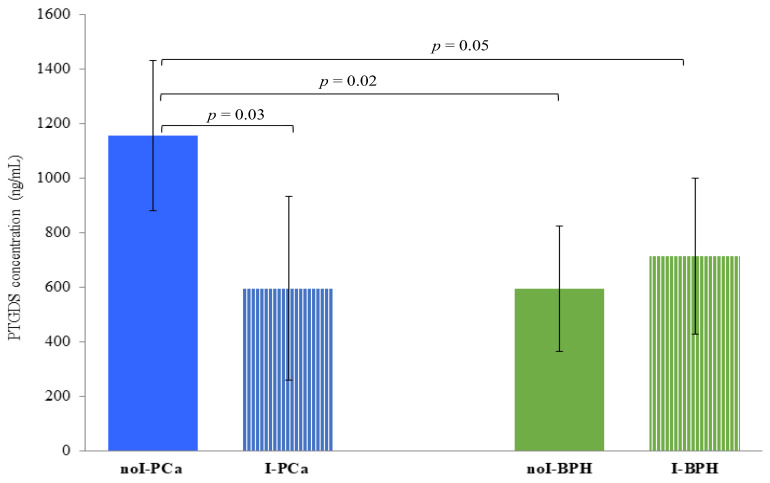
PTGDS quantification by ELISA. Protein concentrations are expressed as mean ± SD. Significant comparisons are highlighted by connecting lines, each showing the *p*-value obtained by Student’s *t*-test (*p* ≤ 0.05 are considered as statistically significant).

The urinary concentration of PTGDS was significantly higher in noI-PCa (1156.8 ± 276.2 ng/mL) compared to: I-PCa (595.6 ± 338.3 ng/mL) (*p* = 0.03), noI-BPH (712.6 ± 285.8 ng/mL), (*p* = 0.02) and I-BPH (593.2 ± 229.3 ng/mL) (*p* = 0.05) (Figure 4). Conversely, no significant differences were found between I-PCa and I-BPH, and I-BPH vs. noI-BPH (*p* > 0.05).

## 4. Discussion

Currently, the gold standard for the detection of clinically significant PCa is MRI-targeted or standard transrectal prostate biopsy [23]. Serum PSA lacks the specificity to discriminate between benign prostatic diseases (such as BPH and prostatitis) and PCa, being organ rather than cancer-specific [24,25]. Hence, there is a reasonable need for novel and unambiguous biomarkers of clinical utility for PCa diagnosis, prognosis, and beneficial treatment strategies [26]. Proteomics proves to be a promising approach for protein biomarkers discovery aimed at improving the management of PCa patients [27]. Furthermore, samples obtained through non-invasive procedures, such as urine, are a suitable target for proteomics [28].

In this study, we analyzed the urinary proteome of patients with PCa and BPH, addressing, for the first time to our knowledge, the role of inflammation as a potential confounding element. We revealed both quantitative and qualitative differences in the urinary protein content between PCa and BPH. Particularly, PCa samples showed a significantly lower protein content than BPH, regardless of inflammation. This could be due to the disease-related modifications of the molecular pathways that cause the adjustment of the metabolic profile observed in PCa and promote the adaptability of the prostate cells to the cancer microenvironment. According to the literature, the changes occurring in PCa can affect the entire central metabolism, and it seems that the prostate has a unique metabolism not found in other types of tissues [29]. When the same disease was compared, taking into account the presence or absence of inflammation, total protein content was significantly lower in I-PCa vs. noI-PCa, while resulting in significantly higher in I-BPH vs. noI-BPH. It is important to underline that inflammation is a pivotal process associated with benign and malignant prostate diseases [30]. Inflammation activates hyperproliferative systems in BPH and creates an appropriate microenvironment for cancer growth and progression [31]. Consequently, this study was performed considering the inflammatory process as a common denominator of both conditions.

Qualitative variations in protein expression were detected by 2-DE and LC-MS/MS analysis through the comparison among the groups. The comparison of the urinary proteome between PCa and BPH, without taking into consideration the presence/absence of inflammation, showed nine deregulated proteins in PCa patients. Specifically, AMBP and SAP3 were found to be upregulated, while AACT, A1BG, TRFE, A1AT, HPT, APOA1 and TTHY resulted in significantly downregulated. As inflammation can affect potential PCa biomarkers in proteomic research [18], PCa and BPH were compared, excluding samples with evident histological signs of inflammation (noI-PCa vs. noI-BPH). In this case, both AMBP spots and SAP3 were increased in PCa, but only AACT, A1BG, A1AT, and APOA1 were decreased. TRFE was not detectable, while HPT and TTHY showed no significant expression difference. Remarkably, in the absence of inflammation was identified the PTGDS protein in PCa samples was not detected in the previous comparison, namely in PCa and BPH groups regardless of inflammation.

AMBP is an acute phase protein (APP) previously revealed as a promising diagnostic biomarker for PCa [32]. Moreover, it has already been proven to be a potential biomarker of other cancer types, such as non-small-cell lung carcinoma [33] and pancreatic cancer [34]. SAP3 is a ganglioside with supported roles in mediating tumor-induced growth and progression [35] and cancer cell migration, as reported by a proteomic analysis of the breast cancer secretome [36]. It is proven that, in tumor cells, the expression levels of gangliosides positively or negatively regulate the signaling of cancer cells, promoting the malignancy of the disease [37]. SAP3 is also implicated in lipids transport. It is well established that lipid metabolism has a key role in PCa progression due to its interactions with androgens and its close involvement in the interactivity between immune and tumor cells [38]. The carcinomic prostate tissue becomes dependent on the use of lipids to survive and proliferate; this could explain the higher expression of SAP3 revealed in PCa vs. BPH. Both AMBP and SAP3 were found to increase also in our previous proteomic study conducted on urine samples from PCa patients with different risks of cancer progression [39]. The glycoprotein AACT is a serine protease inhibitor involved in the acute phase response and proteolysis (UniProtKB). Its dysregulation and glycosylation levels are associated with tumor progression and recurrence [40]. A1BG, another glycoprotein belonging to the immunoglobulin superfamily, was found overexpressed by proteomics in various forms of cancer, such as pancreatic ductal adenocarcinoma [41], cervical intraepithelial neoplasia [42] and bladder cancer [43]. Additionally, A1BG has been found to be associated with tumor heterogeneity and malignancy in an animal model of breast cancer [44]. Recently, its key role in tumorigenesis has been confirmed, suggesting that A1BG could be a promising target for cancer diagnosis, prognosis, and therapy [40]. Regarding A1AT, its high levels have been positively correlated with unfavorable clinical outcomes in cancer [45]. In support of our findings, Dong and co-authors reported a close association between urinary glycoproteins, including A1AT, and an aggressive form of PCa [46]. Furthermore, the higher expression of APOA1 in BPH versus PCa was previously shown in other proteomic studies, thus consolidating our current results [9,32,47].

To evaluate the changes induced in the urinary proteome by inflammation, PCa and BPH with and without inflammation were further compared with each other. In I-PCa vs. noI-PCa comparison, AMBP, SAP3 and AACT appeared under-expressed, while A1BG, A1AT and HPT were overexpressed. TRFE was confirmed not detectable, endorsing the result obtained from the previous comparison (noI-PCa vs. noI-BPH), together with APOA1 and TTHY. On the contrary, PTGDS was found downregulated in I-PCa samples, so it is reasonable to infer that its detection is sensibly affected by the presence of inflammation. The ELISA test confirmed this finding, as PTGDS concentration resulted significantly different only in PCa free of inflammation. Accordingly, PTGDS cannot be considered a candidate biomarker of PCa due to its influence on inflammation. Besides, in our previous study, this protein was identified in the urine of PCa patients with low, intermediate, and high progression risk, so it cannot even be considered an index of tumor aggressiveness [39]. Finally, AMBP, SAP3 and PTGDS were confirmed downregulated, and A1BG, A1AT, and HPT were proved to be upregulated in I-BPH vs. noI-BPH, like the previous comparison concerning PCa. Particularly, HPT was found downregulated in the PCa group in the first comparison (PCa vs. BPH), while the same comparison in the absence of inflammation was not significant. Then, HPT was found to have a significant increase in both conditions in the presence of inflammation, so it cannot be a feasible biomarker of PCa but rather a protein characterizing the urinary proteome of both diseases when present inflammation. Likewise, high levels of HPT were also evidenced in serum samples from PCa and BPH in the presence of inflammation [18], strengthening the assumption that this is more an inflammation-linked protein rather than a disease-associated protein. Peculiarly, the 2 “negative” acute phase proteins TRFE and TTHY, not detectable in previous comparisons, and HEMO, identified exclusively in this comparison, resulted in an increase in the I-BPH group. So, we can assume that these proteins could be associated with the benign disease rather than with the PCa, as well as with the presence of inflammation. Notable, HEMO, a “positive” APP, was also increased in the serum of BPH patients compared to PCa patient groups [18]. Moreover, consistent with our results, HEMO was found to be downregulated in the urine of PCa vs. BPH in a comparative proteomic analysis by Devalieva et al. [48]. In agreement, it is today widely recognized that high-grade prostatic inflammation is significantly more common in subjects with BPH rather than in those with PCa, contributing to the promotion of the disease development [49].

In summary, only five proteins, namely AMBP, SAP3, AACT, A1BG, and A1AT, could be rightly considered potential PCa biomarkers, as they are not affected by the inflammatory process. Moreover, they could discriminate cancer from BPH. Some of these proteins have already been reported in the literature as possible targets of PCa [32,39,46], while others are thought to be promising biomarkers for different types of malignant diseases [33,34,41,42,43,44]. In the future, once validated and endorsed for clinical practice, these new biomarkers could be proposed as a complementary analysis after a PSA-positive scenario to differentiate between PCa and BPH. Furthermore, this set of targeted urinary proteins could be combined in a multi-panel assay to develop a routine diagnostic test for the screening of PCa. Otherwise, TRFE, TTHY and HEMO were found to be closely associated with inflammation and BPH rather than cancer, and therefore, they are unlikely candidate biomarkers for PCa. A similar result was obtained for APOA1, as its expression was linked to BPH. Additionally, HPT results are primarily related to inflammation in both PCa and BPH and, as such, not a likely candidate marker of PCa. Finally, PTGDS expression was affected by the presence of inflammation.

This study has some limitations, including the small sample size in each subgroup. On the other hand, this is a preliminary proteomic investigation conducted on urine samples considering, for the first time, the possible interference of inflammation during the search for protein biomarkers. Future studies with a larger sample size will be needed to validate these initial results. Secondly, only the most questionable protein, PTGDS, was further verified/quantified by a complementary method, i.e., the ELISA test, which has the benefit of being the gold standard procedure to confirm potential protein biomarkers in urine thanks to its high throughput, simplicity, specificity, and sensitivity [15].

## 5. Conclusions

Considering the presence of inflammation as a real confounding element, a promising pattern of diagnostic biomarkers of PCa was identified in urine samples by a proteomic approach. A reliable urinary profile of PCa-associated protein biomarkers, combined with clinical and histopathological information, could represent an integrative diagnostic tool for early cancer screening, ensuring the best selection of patient candidates for biopsy, as well as enhancing patient stratification or setting up a personalized therapeutic strategy, with the potential to improve the clinical management of PCa.

## Figures and Tables

**Table 1:** Clinical data of selected patients.

Groups	Age (Years) Median (Range)	Grade Group	PSA (ng/mL) Median (Range)	PSA Comparisons
PCa (*n* = 30)	68	G < 7 (*n* = 14)	5.80	PCa vs. BPH, *p* = 0.03
	(59–73)	G ≥ 7 (*n* = 16)	(0.80–34.36)	
noI-PCa (*n* = 10)	68.5	G < 7 (*n* = 5)	5.95	noI-PCa vs. noI-BPH, *p* = 0.02
	(62–73)	G ≥ 7 (*n* = 5)	(4.49–34.00)	
I-PCa (*n* = 20)	67	G < 7 (*n* = 9)	5.49	I-PCa vs. I-BPH, *p* > 0.05
	(59–73)	G ≥ 7 (*n* = 11)	(0.80–34.36)	I-PCa vs. noI-PCa, *p* > 0.05
BPH (*n* = 30)	68	-	3.60	
	(59–81)	-	(0.20–25.00)	
noI-BPH (*n* = 11)	67	-	3.65	
	(59–77)	-	(0.20–6.80)	
I-BPH (*n* = 19)	69	-	3.60	I-BPH vs. noI-BPH, *p* > 0.05
	(60–81)	-	(0.40–25.00)	

Statistical analysis was performed by Student’s *t*-test (*p* ≤ 0.05 is considered statistically significant).

**Table 2:** Protein concentration of urinary pools.

Groups	Concentration (μg/μL)	Group Comparisons	*p*-Value
Pca	1.57 ± 0.48	Pca vs. BPH	*p* < 0.001
noI-Pca	1.74 ± 0.49	noI-Pca vs. noI-BPH	*p* ≤ 0.05
I-Pca	1.49 ± 0.26	I-Pca vs. I-BPH	*p* ≤ 0.05
		I-Pca vs. noI-PCa	*p* > 0.05
BPH	4.06 ± 0.57		
noI-BPH	2.72 ± 0.34		
I-BPH	4.77 ± 1.22	I-BPH vs. noI-BPH	*p* ≤ 0.05

Protein content is expressed as mean ± SD. Statistical analysis was performed by Student’s *t*-test.

**Table 3:** Urinary proteins identified by ESI-Q-ToF-MS/MS analysis.

Entry Name ^a^	Protein Name ^b^	Acc. No. ^c^	MW (Da) ^d^	Fold-Change of Protein Expression ^e^			
				PCa vs. BPH ^e1^	noI-PCa vs. noI-BPH ^e2^	I-PCa vs. noI-Pca ^e3^	I-BPH vs. noI-BPH ^e4^
AMBP^1^	Alpha-1-microglobulin	P02760	39,886	+4.40	+2.39	−1.51	−2.28
AMBP^2^	Alpha-1-microglobulin	P02760	39,886	+2.06	+2.70	−1.64	−1.83
SAP3	Ganglioside GM2 activator	P17900	21,281	+3.80	+2.18	−1.53	−2.09
AACT^1^	Alpha-1-antichymotrypsin	P01011	47,792	−7.91	−5.39	−1.77	+4.23
AACT^2^	Alpha-1-antichymotrypsin	P01011	47,792	−1.50	/	−2.78	+3.99
A1BG	Alpha-1-beta-glicoprotein	P04217	54,790	−5.44	−2.60	+4.50	+6.69
TRFE	Serotransferrin	P02787	79,294	−2.18	ND	ND	+2.14
A1AT^1^	Alpha-1-antitrypsin	P01009	46,878	−6.02	/	/	+2.19
A1AT^2^	Alpha-1-antitrypsin	P01009	46,878	−6.31	−1.91	+2.11	+6.35
A1AT^3^	Alpha-1-antitrypsin	P01009	46,878	−2.57	ND	+1.75	+2.53
A1AT^4^	Alpha-1-antitrypsin	P01009	46,878	−1.50	ND	+1.86	/
A1AT^5^	Alpha-1-antitrypsin	P01009	46,878	−10.90	−2.55	+3.56	+10.3
A1AT^6^	Alpha-1-antitrypsin	P01009	46,878	−2.32	−2.30	+10.8	+1.61
HPT^1^	Haptoglobin	P00738	45,861	−3.34	/	+2.27	+2.16
HPT^2^	Haptoglobin	P00738	45,861	−2.02	/	+1.61	+3.11
APOA1^1^	Apolipoprotein A1	P02647	30,759	−6.67	/	ND	/
APOA1^2^	Apolipoprotein A1	P02647	30,759	−10.40	−3.68	ND	/
TTHY	Transthyretin	P02766	15,991	−3.45	/	ND	+3.46
PTGDS	Prostaglandin-H2 D-isomerase	P41222	21,243	ND	+6.53	−2.79	/
HEMO	Hemopexin	P02790	52,385	ND	ND	ND	+2.52

^a^ Protein entry names from UniProtKB database (all with extension -HUMAN), corresponding to those reported in proteins maps (Figure 2); ^b^ Protein complete names; ^c^ Protein accession number from UniProtKB database; ^d^ MW, theoretical molecular weight (Da, Dalton); ^e^ Fold-change of protein expression among groups, (+) increased expression, (−) decreased expression in ^e1^ PCa vs. BPH, ^e2^ noI-PCa vs. noI-BPH, ^e3^ I-PCa vs. noI-PCa, ^e4^ I-BPH vs. noI-BPH. ND: protein spot not detectable. (/): not significant comparison (<1.5).

## Data Availability

All data considered in this study are reported in this article.

## Abbreviations

A1AT	Alpha-1-antitrypsin
A1BG	Alpha-1-beta-glicoprotein
AACT	Alpha-1-antichymotrypsin
AMBP	Alpha-1-microglobulin
APOA1	Apolipoprotein A1
APPs	Acute phase proteins
BPH	Benign prostatic hyperplasia
2-DE	Two-dimensional gel electrophoresis
H&E	Hematoxylin and eosin stain
HEMO	Hemopexin
HPT	Haptoglobin
I-BPH	BPH group with inflammation
I-PCa	PCa group with inflammation
noI-BPH	BPH group without inflammation
noI-PCa	PCa group without inflammation
PBx	Prostate needle biopsy
PCA	Prostate Cancer
PSA	Prostate-specific antigen
PTGDS	Prostaglandin-H2 D-isomerase
SAP3	Ganglioside GM2 activator
TRFE	Serotransferrin
TTHY	Transthyretin
